# Physical and mechanical therapies for lower limb symptoms in children with Hypermobility Spectrum Disorder and Hypermobile Ehlers-Danlos Syndrome: a systematic review

**DOI:** 10.1186/s13047-018-0302-1

**Published:** 2018-11-07

**Authors:** Benjamin Peterson, Andrea Coda, Verity Pacey, Fiona Hawke

**Affiliations:** 1 School of Health Sciences, Faculty of Health and Medicine, University of Newcastle, Central Coast Campus, Ourimbah, NSW 2258 Australia; 2 Faculty of Medicine and Health Sciences, Department of Health Professions, Macquarie University, Sydney, 2109 Australia; 3 Institute of Endocrinology and Diabetes, The Children’s Hospital at Westmead, Westmead, NSW 2145 Australia

**Keywords:** Joint hypermobility syndrome, Lower limb, Foot, Children

## Abstract

**Background:**

Hypermobility Spectrum Disorder and Hypermobile Ehlers Danlos Syndrome are two common heritable genetic disorders of connective tissue. Both conditions are characterised by excessive joint range of motion and the presence of musculoskeletal symptoms, and are associated with joint instability, motion incoordination, decreased joint position sense, and musculoskeletal pain. Hypermobility Spectrum Disorder is the new classification for what was previously known as Joint Hypermobility Syndrome. This systematic review evaluates the evidence for physical and mechanical treatments for lower limb problems in children with Hypermobility Spectrum Disorder and Hypermobile Ehlers Danlos Syndrome.

**Methods:**

MEDLINE, EMBASE, Cochrane Central Register of Controlled Trials, PUBMED and CINAHL were searched to October 2017 for randomised controlled trials (RCT) and quasi-RCTs evaluating physical and mechanical interventions for lower limb problems in children with hypermobility. Two authors independently screened studies for eligibility for inclusion and three review authors independently assessed risk of bias of included studies. One author extracted and analysed statistical data, which were checked by a second author.

**Results:**

Two RCTs including a total of 86 participants were eligible for inclusion. Trials evaluated differences between generalised versus targeted physiotherapy programs and between performing knee extension exercises to the neutral versus hypermobile range. There was no clear benefit of any of the physical therapies evaluated.

**Conclusion:**

There is very limited evidence to guide the use of physical and mechanical therapies for lower limb problems in children with Hypermobility Spectrum Disorder and Hypermobile Ehlers Danlos Syndrome. Mechanical therapies have not been evaluated in RCTs and results of the two RCTs of physical therapies do not definitively guide physical therapy prescriptions. Current studies are limited by small sample sizes and high attrition rates. No physical therapy has been compared to a sham intervention no intervention or no intervention, so overall effectiveness is unknown.

## Background

Hypermobility Spectrum Disorder and Hypermobile Ehlers Danlos Syndrome (hEDS) are two of the most common heritable genetic disorders of connective tissue. They are characterised by excessive joint range of motion and the presence of musculoskeletal symptoms [1]. Hypermobile Ehlers Danlos Syndrome is also characterized by phenotypical features, which differentiate it from Hypermobility Spectrum Disorder [2]. The aetiology of Hypermobility Spectrum Disorder and hEDS is proposed to be associated with abnormalities of fibrous protein genes that encode collagen, elastin and fibrillin [3]. Recent evidence suggested that non-collagenous extracellular matrix proteins, such as tenascin X, may have been involved in some cases [4]. Hypermobility Spectrum Disorder is the new classification for what was previously called Joint Hypermobility Syndrome, which refers to hypermobility of multiple joints, in the absence of a well-defined syndrome [5, 6]. hEDS is the new classification of what was previously called Ehlers-Danlos syndrome-hypermobility type, and the updated diagnostic criteria are now much tighter [2, 5]. The prevalence of Joint Hypermobility Syndrome in children was estimated to range from 5 to 18% [7, 8]. Hypermobility is more common in girls than boys, and prevalence varies with ethnicity and reduces with age [9].

Hypermobility Spectrum Disorder and hEDS involve joint hypermobility and other musculoskeletal impairments such as activity-related pain [10], joint instability [11], muscle weakness, poor balance, motion incoordination [12] and altered gait [3, 11]. Previously, the most commonly used and accepted diagnostic system for JHS was the Brighton criteria [13], although this tool was designed for use in adult populations, and has not been validated in children. The Villefranche criteria were used for diagnosing Ehlers-Danlos syndrome-hypermobility type, and experts previously suggested that EDS-HT was clinically indistinguishable from JHS [14].

As the new classifications have not been used historically in research, the terms Joint Hypermobility Syndrome and Ehlers-Danlos syndrome-hypermobility type will be used in this review only when referring to previous research and included trials.

Children with hypermobility account for up to one quarter of referrals to physiotherapy from paediatric rheumatology clinics [15]. Lower limb pain and instability, particularly at the knee joint, are the most common musculoskeletal complaints in children with hypermobility [16].

Clinicians commonly use physical therapies (such as strengthening, stretching, and co-ordination and balance exercises) [17] and mechanical therapies (such as splints or orthoses) to treat lower limb pain in children with Hypermobility Spectrum Disorder and hEDS Whilst these treatments address deficits identified by previous research, including reduced joint kinesthesia, joint proprioception, and muscle torque in children with hypermobility [11], the research evaluating their effectiveness has not been systematically collated, and individual reports can be difficult for clinicians to access and appraise. As such, many health professionals are unsure of best clinical practice. While some systematic reviews have evaluated interventions for lower limb symptoms associated with hypermobility, these included studies other than randomised controlled trials (RCT), and included both children and adults [17, 18].

## Objective

To systematically review randomised and quasi-randomised trials evaluating non-invasive physical and mechanical therapies in the management of lower limb symptoms in children with hypermobility.

## Methods

The protocol for this systematic review was published prior to performing the study [19]. No variations to the protocol were made in the conduct of the review.

### Types of studies

All RCTs and quasi-RCTs of physical and mechanical therapies for lower limb problems in children with hypermobility were eligible for inclusion. Trials focusing on prevention of hypermobility-related lower limb problems were excluded. A trial could be included regardless of whether or not participants were blinded to their intervention.

### Types of participants

Trials of children age 0–17 years with hypermobility and lower limb symptoms were included. Children could have been diagnosed with JHS or Ehlers-Danlos Syndrome (hypermobility type), or ‘hypermobility’ described as being symptomatic. From our knowledge of the literature, it was expected that JHS would be diagnosed using the Brighton criteria, and Ehlers-Danlos Syndrome (hypermobility type) using the Villefranche criteria, though these diagnostic criteria were not essential for inclusion. Other hypermobility described as being symptomatic was eligible for inclusion. Trials of asymptomatic hypermobility and other heritable disorders of connective tissue (e.g. Osteogenesis Imperfecta, Marfan Syndrome) were excluded. Lower limb symptoms included any pathology of the gluteal and femoral regions, and any pathology distal to that, including pathology of the ankles and feet [20].

### Types of settings

All settings were eligible for inclusion.

### Types of intervention

Any non-invasive mechanical or physical therapy for lower limb problems was eligible for inclusion. Mechanical interventions include braces, splints, footwear and orthotic devices. Physical interventions include massage, stretching, and strengthening exercises. All other mechanical and physical interventions were also eligible for inclusion. Trials of drug therapies, surgery and invasive interventions (e.g. dry needling) were ineligible. Trials were excluded if the effect of the mechanical or physical intervention could not be isolated (e.g. if the mechanical or physical therapy was provided in combination with other therapies).

### Primary outcome

Quantifiable and validated measures of health-related quality of life (for example, PedsQL Measurement Model) [21].

### Secondary outcomes

Any validated quantifiable measure of pain (for example: the Paediatric Pain Questionnaire) [22]; disability or functional ability, or both; hypermobility-related injuries (for example ankle sprains); participant satisfaction with intervention; fatigue and adverse events. Tools validated for use in children were included even when not specifically validated for hypermobility.

### Searching for studies

Electronic searches were performed on 31st of October 2017. Search terms for OVID MEDLINE (from January 1966 to 31/10/2017) were:

1. Randomized controlled trial.pt.
2. Controlled clinical trial.pt.
3. Randomi?ed.ti,ab
4. Placebo.ti,ab
5. Randomly.ti,ab
6. Trial.ti,ab
7. Group?.ti,ab
8. Allocate?.ti,ab
9. 1 or 2 or 3 or 4 or 5 or 6 or 7 or 8
10. Exp animals/not humans
11. 9 not 10
12. Hypermobi*.tw
13. Ehlers-danlos.tw
14. Beighton.tw
15. Brighton.tw
16. 12 or 13 or 14 or 15
17. Child*.tw
18. P?ediatric.tw
19. Adolescent.tw
20. Juvenile.tw
21. 17 or 18 or 19 or 20
22. 11 and 16 and 21

This was adapted to suit the following databases: Cochrane Central Register of Controlled Trials (CENTRAL) (The Cochrane Library, Issue 10 2017); EMBASE (January 1980 to October 2017); CINAHL (January 1982 to October 2017); and PUBMED (January 1966 to October 2017). No language or publication restrictions were applied and authorship/ results were not masked. Reference lists of included studies were screened for other eligible studies. Researchers and corresponding authors of eligible studies were contacted by email to help identify other published or unpublished potentially eligible articles.

Two authors (BP and either AC or FH) assessed titles and abstracts of studies retrieved by the electronic searches and retrieved full-text versions of all potentially eligible studies. These were assessed by two authors (BP and either AC or FH) for inclusion. Disagreements between BP and AC were mediated by FH. There were no disagreements between BP and FH. Study authors were contacted as required to determine study eligibility.

### Data extraction

Data were extracted using standardised pilot-tested forms by one reviewer (BP) and were checked by another reviewer (AC). Extracted data included: study design, inclusion and exclusion criteria, recruitment procedures, setting, interventions, outcome measures, follow up duration, power calculation, baseline characteristics, outcome data, and funding source. Corresponding authors were contacted for more information as required.

### Risk of bias appraisal

The risk of bias of included studies was independently assessed by BP, AC and FH using the following criteria described in the Cochrane Handbook for Systematic Reviews of Interventions [23]:

- sequence generation;
- concealment of allocation;
- blinding or participants
- blinding of personnel who administered the intervention;
- blinding of outcome assessors’
- incomplete data;
- selective outcome reporting;
- other sources of bias

Each criterion was assigned a judgement of ‘high risk’, ‘low risk’ or ‘unclear risk’. All disagreements in the assessment of risk of bias were resolved by discussion by BP, AC and FH.

### Data analysis

Data for different types of interventions and different lower limb problems were analysed separately in Review Manager 5 [24] following Cochrane Handbook guidelines [23]. Data were entered by one reviewer (FH) and checked by another (BP). Where data were not appropriate for analysis in Review Manager 5, additional data were requested from corresponding authors. In the event that corresponding authors did not provide appropriate data as requested by email, the results were reported as they appeared in the original trial publication. Continuous data that were analysed in Review Manager 5 were analysed using a fixed effect model and the inverse variance method with mean difference effect measure with 95% confidence intervals (CI). Analyses of dichotomous data were to report odds ratios (OR) with 95% CI, though this was not required. If meta-analysis was performed, sensitivity analysis was to exclude papers that did not conceal allocation sequence, and outliers if the reason for the errant result was evident, though this was not required.

## Results

### Description of studies

Electronic searching retrieved 520 titles and abstracts following removal of duplicates. Of these, 24 were identified as being potentially relevant [3, 15–18, 25–43]. Upon reading full texts, 21 articles were excluded (14 were not RCTs or quasi-RCTs [3, 15–18, 25–33], 4 included an inappropriate age range [34–37], 2 did not address a specific lower limb complaint [38, 39], and 1 reported non quantifiable measures [40]). Authors of the remaining three trials were contacted to determine eligibility. Of these, one was excluded as research was ongoing [41].

**Fig. 1:**
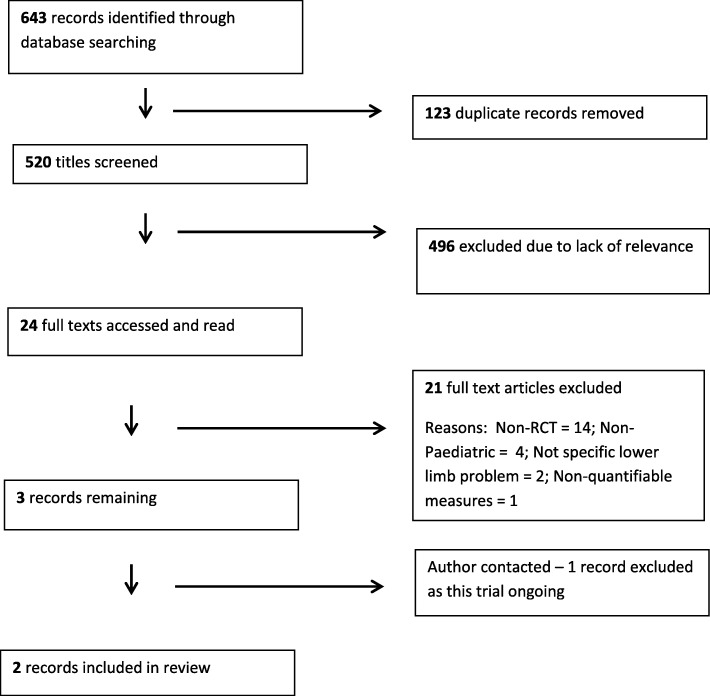
PRISMA Flow Diagram

Two studies were eligible for inclusion [42, 43]. Reference lists of both included trials were screened for other potentially eligible trials and known researchers in the field were contacted by email to identity other potentially relevant studies. No additional studies were identified. Fig. 1 presents the PRISMA flow diagram.

### Included studies

Both included studies were parallel-group randomised controlled trials published in peer-reviewed journals between 2010 and 2012 [42, 43]. Table 1 summarises characteristics of included studies.

**Table 1:** Summary – Characteristics of included studies 643 records identified through database searching

Characteristic	Pacey et al. (2013)	Kemp et al. (2010)
Lower Limb Problem	Knee pain of no known aetiology	Arthralgia for at least 3 months preceding
Intervention Groups	1. Physiotherapist supervised 8-week physical therapy program, including exercises to address muscle strength and motion control performed into the neutral range of knee extension 2. Physiotherapist supervised 8-week physical therapy program, including exercises to address muscle strength and motion control performed into the full range of knee hyperextension	1. Physiotherapist supervised 6-week generalised physiotherapy programme, aimed at improving muscle strength and fitness 2. Physiotherapist supervised 6-week targeted physiotherapy programme, specifically addressing functional stability of symptomatic joints
N participants	29	57
Age Range	7–16 years	7–16 years
Recruitment Source	Children referred to The Children’s Hospital at Westmead’s Physiotherapy, Sports Medicine, Orthopaedic Knee, Connective tissue Dysplasia and Rheumatology clinics (January 2007 – February 2011)	Children treated at Alder Hey Children’s Hospital NHS Foundation, Liverpool (June 2004 – May 2007)
Follow-up Period	8 weeks	5 months
Outcomes	1. Pain • Child-reported mean knee pain (over one week) (VAS) • Parent-reported maximum knee pain (over one week) (Parental-VAS) 2. Patients global impression of change (PGIC) 3. Functional ability (CHAQ) ^a^ 4. Quality of life (Child Health Questionnaire) 5. Functional impairment (Mean quadriceps and Hamstring Strength) 6. Functional ability (Number of flights of stairs climbable in 2 min)	1. Pain • Improvements in child’s pain assessment score (VAS for > 11 years of age/Wong-Baker Faces < 11 years of age) • Parent’s assessment of child’s pain (Parental-VAS) 2. Parents global evaluation of the impact of their child’s hypermobility over the previous week (Global-VAS) 3. Functional Ability (CHAQ)^a^ 4. Functional ability (Six-minute shuttle level assessment) ^b^

^a^Childhood Health Assessment Questionnaire

^b^Baseline and midpoint only

### Participants

A total of 86 participants (48 males and 38 females) were included in the two trials [42, 43]. All participants in both trials were diagnosed using the Brighton criteria. The mean age of participants in the trial by Kemp et al. (2010) was 10.88 years (SD = 2.5) and in the trial by Pacey et al. (2013) was 12.04 years (SD = 2.93).

### Lower limb problems

Both trials assessed arthralgia associated with JHS. The trial by Kemp et al. (2010) included participants presenting with arthralgia for at least the previous three months. The trial by Pacey et al. (2013) included participants presenting with JHS-associated knee pain.

### Types of interventions

#### Physical therapies

The trial by Kemp et al. (2010) assessed the effectiveness of generalised vs targeted physiotherapy for improving child-rated and parent-rated subjective pain, health-related quality of life, and functional ability. Participants attended six sequential weekly 30-min appointments where the allocated physical therapy intervention was administered under the supervision of a senior physiotherapist. The treating therapist was blind to demographic data, diagnostic hypermobility criteria, symptom scores and assessment of joint range, muscle strength and fitness. Generalised physiotherapy involved the prescription of standardized general exercises aimed at improving muscular strength and fitness, including bunny hops, shuttle-runs, squat-thrusts, sit-to-stand, step-ups and star jumps. Participants in this treatment group were provided with a take-home exercise programme to perform daily, based on their achievement at physical therapy sessions. Normal activities, including return to sport were also encouraged. Targeted physiotherapy involved the prescription a step-wise standardised physical therapy exercise programme which specifically addressed functional stability re-training of symptomatic joints. Exercises were performed for pre-determined time intervals or repetitions which were increased as each exercise was achieved more easily. Participants in the targeted physical therapy group were given a home exercise programme tailored to their level of postural control. Home exercise prescriptions were to be performed daily, within pain-free limits.

The trial by Pacey et al. (2013) compared the effectiveness of an eight-week physical therapy program which addressed muscle strength and joint control with exercises performed into the neutral range of knee extension versus hypermobile range of knee extension. Outcomes were pain, health-related quality of life and functional ability. Treatment allocation followed a baseline period of no intervention of at least two weeks duration. Participants were given three to five exercises within their individual capacity to complete a minimum of five times per week and advised that each session should not take more than 30 min.

#### Mechanical therapies

Neither of the included randomised controlled trials assessed the effectiveness of mechanical therapies in managing lower limb problems in hypermobility.

### Risk of Bias

Risk of bias assessment is presented in Table 2.

**Table 2:** Risk of bias assessment summary

Risk of Bias				
Domain	Pacey et al. (2013)	Evidence	Kemp et al. (2010)	Evidence
Sequence Generation	Low risk	‘The simple randomisation list was generated in a 1:1 ratio using a computer-generated sequence by a person independent of the research group.’	Low risk	‘The randomization list was generated in a 1: 1 ratio using a computer-generated sequence with random variable block size of four and six.’
Allocation Concealment	Low risk	‘Treatment allocation was concealed in a sealed, opaque, sequentially numbered envelope which was opened by the treating physiotherapist just prior to the participant’s first physiotherapy session.’	Low risk	‘Treatment allocation was concealed by placing an allocation card between two blank cards in a sealed, opaque, sequentially numbered envelope.’
Blinding of Participants, Personnel and Outcome Assessors	High risk	Participants: Not blinded Personnel: Not blinded Outcome assessors: for participant-rated outcomes, not blinded. For other measures, outcome assessor was blinded. ‘Following the 8 week intervention, participants underwent a third assessment by an assessor blinded to treatment allocation.’	High risk	Participants: Not blinded Personnel: Not blinded Outcome assessors: for participant-rated outcomes, not blinded. For other measures, outcome assessor was blinded. ‘All physiotherapy assessments (at baseline, mid-point assessment and final follow-up) …. were conducted by one senior physiotherapist assessor (I.R.); patients and treating physiotherapist (S.K.) were asked not to divulge the allocated treatment to the assessing physiotherapist.’
Incomplete outcome data	Unclear	One participant was lost to follow up from the ‘training in hypermobile range’ group. The participant was unable to be contacted so the reason for loss to follow up is not known.	Low risk	Missing outcome data were balanced in numbers across intervention groups, with similar reasons for missing data across groups. Reasons included: repeated non-attendance, successful rehabilitation, changes in family circumstances and requirement for further investigation.
Selective outcome reporting	Unclear	Insufficient information to permit judgment.	Unclear	Insufficient information to permit judgment.
Other sources of bias	Low risk	None identified.	Low risk	None identified.

### Outcomes

Pain severity was assessed in both trials. The trial by Pacey et al. (2013) employed the 100 mm Visual Analogue Scale (VAS) on which children indicated their level of pain as both an average and maximum rating of pain intensity over the past week. The trial by Kemp et al. (2010) also used the VAS scale to quantify parent-report of child’s pain, which was self-reported by children aged eleven and older. Children below eleven years of age in the Kemp et al. (2010) trial reported pain using the Wong-Baker Faces adaptation of the VAS scale. Physical functioning was also assessed by both authors using the Child Health Assessment Questionnaire (Minimally important difference (MID) for improvement: − 0.188) [44]. The trial by Pacey et al. (2013) assessed functional ability, by measuring the number of flights of stairs participants could climb in two minutes (MID unknown), whereas this outcome measure was assessed by Kemp et al. (2010) via a six-minute shuttle walking test (MID: 54–80 m) [45].

### Effects of interventions

In total, 86 participants were allocated to undergo treatment via physical therapy. In the trial by Pacey et al. (2013), 12 participants were allocated to the hypermobile training group and 14 were allocated to the neutral training group. Four participants withdrew from this trial prior to random allocation, including one who was later diagnosed with osteogenesis imperfecta, thus rendering them ineligible for participation. In the Kemp (2010) trial, 30 participants were allocated to the targeted physical therapy group and 27 participants were allocated to the generalized physical therapy group.

### Data and analysis

In both studies, clinically important baseline differences precluded the use of unadjusted follow-up data in Review Manager 5. Mean change from baseline scores were reported by Kemp et al. (2010) and were analysed in Review Manager 5 for this review. Results are presented in Table 3. The contact author of the trial by Pacey et al. (2013) were asked to provide change-from-baseline standard deviation data for all outcomes included in this review, however, none were provided. Hence, no re-analysis of data were performed for the trial by Pacey et al. (2013). The results from the trial by Pacey et al. (2013) reported in this systematic review reflects the original trial report.

**Table 3:** Targeted physiotherapy versus generalize physiotherapy

Outcome	Targeted physiotherapy		Generalised physiotherapy		Between groups difference
	Mean change (SD)	*n*	Mean change (SD)	*n*	Mean (95% CI)
Baseline to 3 months					
Child’s pain assessment^a^	−25.78 (28.37)	23	−29.75 (38.63)	18	3.97 (−17.31 to 25.25)
Parent’s pain assessment	−19.91 (23.12)	23	−19.64 (23.33)	18	−0.27 (−14.60 to 14.60)
CHAQ	−0.24 (0.54)	23	−0.14 (0.55)	18	−0.10 (− 0.44 to 0.24)
Shuttle-level assessment	2.83 (13.64)	23	0.94 (18.46)	18	1.89 (−8.30 to 12.08)
Baseline to 5 months					
Child’s pain assessment	−21.23 (33.07)	17	−30.64 (37.34)	15	9.41 (−15.17 to 33.99)
Parent’s pain assessment	−21.62 (24.43)	17	−12.13 (22.14)	15	−9.49 (−25.72 to 6.74)
CHAQ	−0.15 (0.27)	17	−0.16 (0.50)	15	0.01 (−0.27 to 0.29)

^a^Measured with VAS/Wong Baker. Lower score desirable for all outcomes but shuttle-level assessment. *CHAQ* Child Health Assessment Questionnaire

These results are presented in Table 4.

**Table 4:** Outcomes after 8 weeks training to neutral range versus hypermobile range of knee extension

Outcome	Neutral range training group (*n* = 14)			Hypermobile range training group (*n* = 11)			Between group differences		Cohen’s D
	Baseline Mean (SD)	8 weeks Mean (SD)	Mean Change	Baseline Mean (SD)	8 weeks Mean (SD)	Mean Change	Mean change difference	95% CI	
Child reported knee pain (mean) ^a^	40.04 (16.59)	20.14 (18.37)	−19.9	38.55 (16.89)	29.36 (17.99)	−9.19	10.71	−7.9 to 29.33	0.61
Child reported knee pain (max)^a^	57.68 (23.12)	35.64 (28.57)	−22.04	53.23 (23.55)	39.18 (27.21)	−14.05	7.99	−14.66 to 30.64	0.31
CHAQ Score	−0.13 (0.44)	−0.01 (0.60)	0.12	0.04 (0.71)	0.05 (0.72)	0.02	0.10	−0.25 to 0.45	0.16
No. flights of stairs/2 min	16.32 (5.00)	20.11 (5.52)	3.79	20.88 (6.69)	20.55 (5.44)	−0.33	−4.12	0.301 to − 8.523	0.73
CHQ Physical Summary^c^	32.01 (11.86)	42.08 (10.81)	10.07	41.61 (14.96)	43.91 (15.05)	2.3	−7.77	−14.99 to -.055^b^	0.59
CHQ Psychosocial Summary^c^	46.35 (12.26)	45.41 (13.49)	−0.94	46.29 (8.95)	54.41 (4.42)	8.12	9.06	2.66 to 15.47^b^	0.83

^a^Using 100 mm VAS Scale; ^b^statistically significant, ^c^A difference of 7 or more points indicates a clinically significant difference [47].*CHAQ*: Child Health Assessment Questionnaire, *N* Newtons. CHQ: Child Health Questionnaire. Lower score desirable for all outcomes but number of flight of stairs/2 min and CHQ summaries

The trial by Pacey et al. (2013) measured the change in outcomes during the baseline control period prior to group allocation. During this period, there were statistically significant improvements in only the parent-reported limitations in emotion and behaviour domain of the CHQ (*p* = 0.02), which resulted in improvement in the Psychosocial Summary of the CHQ (*p* = 0.03). The size of the change was not reported. There were no statistically significant changes for any other outcome measured prior to intervention.

No statistically significant differences were found between targeted and generalised physiotherapy after three or five months. In the trial by Pacey et al. (2013), comparison of exercising knees into the neutral or hypermobile range for eight weeks, no statistically significant differences were found between groups for the outcomes of pain, CHAQ or function measured by number of flights of stairs climbed per two minutes. There were statistically significant differences between neutral knee range and hypermobile knee range training groups for CHQ. The CHQ Physical summary improved more in the neutral knee range training group (mean difference − 7.77; 95% CI: 0.055 to 14.99), however, there were large between-group differences at baseline, which may have influenced these results. The CHQ Psychosocial summary improved more in the hypermobile range training group (mean difference 9.06; 95% CI: 2.66 to 15.47). Pacey et al. (2013) report that the following three individual domains of CHQ significantly favoured the exercise into the hypermobile range group: self-esteem (*p* = 0.03), behaviour (*p* = 0.019) and mental health (*p* = 0.001). Between-group change from baseline standard deviation data for these domains were not reported. None of the individual domains significantly favoured the group exercising into the neutral range of knee extension. The trial by Pacey et al. (2013) noted that self-esteem and mental health domains were significantly lower than Australian normative values at baseline (all *p* < 0.05) [46]. Only the hypermobile training group equalled Australian norms post treatment (self-esteem: hypermobile *p* = 0.84, neutral *p* < 0.05; mental health: hypermobile *p* = 0.53, neutral *p* < 0.05). Differences between pre- or post- training mean scores of either group with Australian norms were not seen in behaviour domains (all *p* > 0.05).

## Discussion

There is little evidence to guide the use of physical and mechanical interventions for lower limb problems in children with hypermobility (currently classified as Hypermobility Spectrum Disorder or hEDS). The evidence that does exist is limited to comparisons of different types of physical therapy from two randomised controlled trials. In both trials JHS was diagnosed with the Brighton Criteria, which is not validated in children. As the new diagnostic criteria for Hypermobility Spectrum Disorder and hEDS have only recently been agreed, they are yet to be validated in children.

When comparing exercising knees to the neutral knee range versus into the hypermobile range, there were no statistically significant differences between groups for the outcomes of pain, function or health status as measured by the Child Health Assessment Questionnaire. There were statistically and clinically significant differences in quality of life measured with the Child Health Questionnaire but these were contradictory. The psychosocial summary favoured the hypermobile exercise range group (mean difference 9.06; 95% CI: 2.66 to 15.47), and the physical summary favoured the neutral knee range group (mean difference 7.77; 95% CI: 0.055 to 14.99). However, this may have been a ceiling effect due to large between-group differences at baseline.

When comparing targeted versus generalised physiotherapy, no statistically significant differences were found between groups after three or five months for any outcome measures. Outcomes included child and parent assessments of pain, health status as measured by the Child Health Assessment Questionnaire, and function measured with a shuttle-run level assessment.

For the outcome of pain, neither study found statistically significant differences between groups. There was no consistent relationship between improvements in pain intensity and improvement in quality of life. In Pacey et al. (2013) comparison of exercising to the hypermobile versus neutral knee range, the size of the mean difference in pain scores between groups points to possible clinical significance favouring exercising to the neutral knee range but this did not reach statistical significance. These data do not provide a clear guide for management of Hypermobility Spectrum Disorder of hEDS and further research with larger samples is required to guide clinical practice. In the study by Kemp et al. (2010), children below eleven years of age reported pain using the Wong-Baker Faces adaptation of the VAS scale. This has not been validated for use in children with hypermobility so results should be interpreted with caution.

In both trials, the sample sizes at study completion were smaller than the estimates required from power calculations. In the trial by Pacey et al. (2013), 25 of the 26 participants who were allocated to an intervention group completed the final outcome measurement (required sample size of 26). In the trial by Kemp et al. (2010) 32 of the 57 (56%) participants allocated to an intervention group completed final outcome assessment (required sample size of 96). In Kemp’s study (2010), the large drop-out rate places the study at high risk of attrition bias, as the small sample size is only one third of that estimated by power calculations. This drastically reduces the ability of the study to detect a clinically important difference where one exists. For future trials of Hypermobility Spectrum Disorder or hEDS in children, researchers should further consider the strategies to maximize follow-up and reduce attrition.

Neither trial evaluated the effectiveness of physical therapies in comparison to no intervention or a sham/placebo intervention for improving quality of life, pain or function in children with lower limb problems in hypermobility. It is therefore not possible to ascertain the effectiveness of physical therapies in general for improving quality of life, pain levels or functional ability in children with Hypermobility Spectrum Disorder or hEDS. A recent physical therapy guideline for JHS and hEDS has highlighted the deficit in size and quality population-specific research in this area, and the need for rigorous, longer-term multi-centre RCTs of physical therapies for individuals with JHS/hEDS [26].

The current evidence from randomised controlled trials of physical therapies for lower limb problems in children with hypermobility is limited to trials evaluating the effectiveness of variations in physical therapy protocols between treatment groups. At present, no randomised controlled trial has evaluated the effectiveness of any mechanical therapies for lower limb problems in children with hypermobility, despite these interventions being used commonly in clinical practice. Such mechanical interventions include foot orthoses, splints, taping and specific footwear (e.g. high-top footwear). Future research should address this important gap in the literature.

This systematic review attempts to collate all available evidence from RCT and quasi-RCT evaluating physical and mechanical therapies for lower limb pathologies in Hypermobility Spectrum Disorder and hEDS. This is limited by the lack of validated diagnostic criteria for Hypermobility Spectrum Disorder and hEDS in children, and the recent change in disease classification. As such, this review includes papers referring to the recently out-dated classification of ‘Joint Hypermobility Syndrome’ and includes papers using diagnostic tools not validated in children. In our search strategy, we hoped ‘hypermobi*’ would capture all relevant papers and included ‘Beighton’ and ‘Brighton’ as safety nets. We acknowledge that including other assessment tools such as the Villefranche, Hospital del Mar criteria and the Lower Limb Assessment score would have increased the scope of the safety net. As our extended searching of reference lists of included studies and contact with authors did not retrieve other relevant studies we are confident in the penetration of our search.

## Conclusion

There is very limited evidence to guide the use of physical and mechanical therapies for lower limb problems in children with Hypermobility Spectrum Disorder or hEDS. Mechanical therapies have not been evaluated in RCTs and results of the two RCTs of physical therapies do not definitively guide physical therapy prescriptions. From the available evidence, there is no clear benefit of performing exercise into the neutral range of knee extension compared with performing the same exercise prescription into the hypermobile range of knee extension, and no clear benefit of a targeted physical therapy program compared with a generalised physical therapy program. Physical therapy prescription, regardless of joint range or type of exercise, may provide benefit in regard to pain intensity, however this has not been adequately established by available research. Future research should validate tools to diagnose Hypermobility Spectrum Disorder and hEDS in children, and to measure outcomes in these populations. Future RCTs should evaluate the effectiveness of the range of available mechanical therapies. Of utmost importance, future trials should be adequately powered and maximize follow-up to detect clinically important effects.

## Abbreviations

CI: Confidence intervals
hEDS: Hypermobile Ehlers-Danlos Syndrome
JHS: Joint Hypermobility syndrome
MD: Mean difference
MID: Minimally important difference
QoL: Quality of life
RCT: Randomised controlled trial
SMD: Standardised mean differences

## References

[1] Bird HA (2007). Joint hypermobility. Musculoskeletal Care.

[2] Malfait F, Francomano C, Byers P, Belmont J, Berglund B, Black J, Bloom L, Bowen JM, Brady AF, Burrows NP, Castori M, Cohen H, Colombi M, Demirdas S, De Backer J, De Paepe A, Fournel-Gigleux S, Frank M, Ghali N, Giunta C, Grahame R, Hakim A, Jeunemaitre X, Johnson D, Juul-Kristensen B, Kapferer-Seebacher I, Kazkaz H, Kosho T, Lavallee ME, Levy H, Mendoza-Londono R, Pepin M, Pope FM, Reinstein E, Robert L, Rohrbach M, Sanders L, Sobey GJ, Van Damme T, Vandersteen A, van Mourik C, Voermans N, Wheeldon N, Zschocke J, Tinkle B (2017). 2017. The 2017 international classification of the Ehlers–Danlos syndromes. Am J med Genet Part C Semin Med Genet.

[3] Fatoye FA, Palmer S, van der Linden ML (2011). Gait kinematics and passive knee joint range of motion in children with hypermobility syndrome. Gait Posture.

[4] Zweers MC, Kucharekova M, Schalkwijk J (2005). Tenascin-X: a candidate gene for benign joint hypermobility syndrome and hypermobility type Ehlers-Danlos syndrome?. Annals of Rheumatic Diseases.

[5] Hypermobility syndromes association: hypermobility disorders – an update for clinicians. http://hypermobility.org/professionals-section/hypermobility-disorders-an-update-for-clinicians. Published March 16, 2017. Accessed 13 May, 2017.

[6] Castori M, Tinkle B, Levy H, Grahame R, Malfait F, Hakim A (2017). A framework for the classification of joint hypermobility and related conditions. Am J Med Genet C Semin Med Genet.

[7] Qureshi AU, Maalik A, Ahmad TM (2010). Relationship of joint hypermobility and musculoskeletal problems and frequency of benign joint hypermobility syndrome in children. J Ayub Med Coll Abbottabad.

[8] Remvig L, Kümmel C, Kristensen J, Boas G, Juul-Kristensen B (2011). Prevalence of generalized joint hypermobility, arthralgia and motor competence in 10-year-old school children. Int Musculoskelet Med.

[9] Jansson A, Saartok T, Werner S (2004). General joint laxity in 1845 Swedish school children of different ages: age- and gender-specific distributions. Acta Paediatr.

[10] Adib N, Davies K, Grahame R (2005). Joint hypermobility syndrome in childhood. A not so benign multisystem disorder?. Rheumatol.

[11] Fatoye F, Palmer S, Macmillan F (2009). Proprioception and muscle torque deficits in children with hypermobility syndrome. Rheumatol.

[12] Schubert-Hjalmarsson E, Ohman A, Kyllerman M (2012). Pain, balance, activity, and participation in children with hypermobility syndrome. Pediatr Phys Ther.

[13] Grahame R, Bird HA, Child A (2000). The revised (Brighton 1998) criteria for the diagnosis of benign joint hypermobility syndrome (BJHS). J Rheumatol.

[14] Tinkle BT, Bird HA, Grahame R (2009). The lack of clinical distinction between the hypermobility type of Ehlers-Danlos syndrome and the joint hypermobility syndrome (a.k.a. hypermobility syndrome). Am J Med Genet A.

[15] Kerr A, Macmillan CE, Uttley WS (2000). Physiotherapy for children with hypermobility syndrome. Physiotherapy.

[16] Pacey V, Tofts L (2015). Adams, et al. quality of life prediction in children with joint hypermobility syndrome. J Paediatr Child Health.

[17] Palmer S, Bailey S, Barker L (2014). The effectiveness of therapeutic exercise for joint hypermobility syndrome: a systematic review. Physiotherapy.

[18] Smith TO, Bacon H, Jerman E (2014). Physiotherapy and occupational therapy interventions for people with benign joint hypermobility syndrome: a systematic review of clinical trials. Disabil & Rehabil.

[19] Hawke F, Peterson B, Gasser J, Pacey V, Coda A (2016). Physical and mechanical therapies for lower limb problems in children with joint hypermobility syndrome: a systematic review protocol. Appl Clin Res, Clin Trials Regul Aff.

[20] Moore KL, Dalley AF, Agur AM, Taylor C, Heise J, Montalbano J (2013). Lower limb. Clinically oriented anatomy.

[21] Varni JW, Seid M, Rode CA (1999). The PedsQL™: measurement model for the pediatric quality of life inventory. Med Care.

[22] Gragg RA, Rapoff MA, Danovsky MB (1996). Assessing chronic musculoskeletal pain associated with rheumatic disease: further validation of the pediatric pain questionnaire. J Pediatr Psychol.

[23] Higgins JP, Green S. Cochrane handbook for systemic reviews of interventions. Version 5.1.0 [updated March 2011]. The Cochrane Collaboration, 2011. Available from http://handbook.cochrane.org.

[24] Review Manager. 5.3 ed. Copenhagen: The Nordic Cochrane Centre, The Cochrane Collaboration 2014.

[25] Engelbert RHH, Scheper MC (2011). Joint hypermobility with and without musculoskeletal complaints: a physiotherapeutic approach. Int Musculoskelet Med.

[26] Engelbert RH, Juul-Kristensen B, Pacey V (2017). The evidence-based rationale for physical therapy treatment of children, adolescents, and adults diagnosed with joint hypermobility syndrome/hypermobile Ehlers Danlos syndrome. Am J Med Genet Part C.

[27] Furlan AD, Imamura M, Dryden T, Irvin E. Massage for low-back pain. Cochrane Database Syst Rev. 2008;4. 10.1002/14651858.CD001929.pub2. 10.1002/14651858.CD001929.pub2 18843627

[28] Junge T, Larsen LR, Juul-Kristensen B (2015). The extent and risk of knee injuries in children aged 9-14 with generalised joint hypermobility and knee joint hypermobility - the CHAMPS-study Denmark. BMC Musculoskelet Disord.

[29] Maillard SM, Adkins D, Haggart E, et al. Physiotherapy management of children with hypermobility: a review of an out-patient self-management exercise programme. Arthritis Rheum. 2010;62:1342. https://acrabstracts.org/wp-content/uploads/2018/06/2010_ACR_ARHP_Abstract_Supplement.pdf.

[30] Marcolin ALV, Cardin SP, Magalhães CS. Muscle strength assessment among children and adolescents with growing pains and joint hypermobility. Brazilian Journal of Physical Therapy / Revista Brasileira de Fisioterapia. 2009; 13(2): 110–5. Available from: Academic Search Complete, Ipswich, MA. Accessed 21 Mar 2016.

[31] Moller A, Masharawi Y (2011). The effect of first ballet classes in the community on various postural parameters in young girls. Phys Ther Sport.

[32] Pacey V, Adams RD, Tofts L (2014). Proprioceptive acuity into knee hypermobile range in children with joint hypermobility syndrome. Pediatr Rheumatol Online J.

[33] Scheper MC, Pacey V, Rombaut L (2017). Generalized hyperalgesia in children and adults diagnosed with hypermobility syndrome and Ehlers-Danlos syndrome hypermobility type: a discriminative analysis. Arthritis Care Res.

[34] Fritz JM, Whitman JM, Childs JD (2005). Lumbar spine segmental mobility assessment: an examination of validity for determining intervention strategies in patients with low back pain. Arch Phys Med Rehabil.

[35] Celestini M, Marchese A, Serenelli A (2005). A randomized controlled trial on the efficacy of physical exercise in patients braced for instability of the lumbar spine. Eura Medicophys.

[36] Keays SL, Mason M, Newcombe PA (2015). Individualized physiotherapy in the treatment of patellofemoral pain. Physiother Res Int.

[37] Sahin N, Baskent A, Cakmak A (2008). Evaluation of knee proprioception and effects of proprioception exercise in patients with benign joint hypermobility syndrome. Rheumatol Int.

[38] Mintz-Itkin R, Lerman-Sagie T, Zuk L (2009). Does physical therapy improve outcome in infants with joint hypermobility and benign hypotonia?. J Child Neurol.

[39] Morrison SC, Ferrari J, Smillie S (2013). Assessment of gait characteristics and orthotic management in children with developmental coordination disorder: preliminary findings to inform multidisciplinary care. Res Dev Disabil.

[40] Birt L, Pfeil M, MacGregor A (2014). Adherence to home physiotherapy treatment in children and young people with joint hypermobility: a qualitative report of family perspectives on acceptability and efficacy. Musculoskeletal Care..

[41] Bale PJ, Easton V, Bacon H (2015). The efficacy of a multidisciplinary intervention strategy for the treatment of benign joint hypermobility syndrome (BJHS) in childhood. A randomised, single Centre parallel group trial (the bendy study). Arch Dis Child.

[42] Pacey V, Tofts L, Adams RD (2013). Exercise in children with joint hypermobility syndrome and knee pain: a randomised controlled trial comparing exercise into hypermobile versus neutral knee extension. Pediatr Rheumatol Online J..

[43] Kemp S, Roberts I, Gamble C (2010). A randomized comparative trial of generalized vs targeted physiotherapy in the management of childhood hypermobility. Rheumatology (Oxford).

[44] Brunner HI, Klein-Gitelman MS, Miller MJ (2005). Minimal clinically important differences of the childhood health assessment questionnaire. J Rheumatol.

[45] Wise R, Brown C (2005). Minimal clinically important differences in the six-minute walk test and the incremental shuttle walking test. COPD.

[46] Waters E, Salmon L, Wake M (2000). The child health questionnaire in Australia: reliability, validity and population means. Aust N Z J Public Health.

[47] Hullmann SE, Ryan JL, Ramsey RR (2011). Measures of general pediatric quality of life: child health questionnaire (CHQ), DISABKIDS chronic generic measure (DCGM), KINDL-R, pediatric quality of life inventory (PedsQL) 4.0 generic Core scales, and quality of my life questionnaire (QoML). Arthritis Care Res (Hoboken).

